# Damage Evaluation of Concrete Column under Impact Load Using a Piezoelectric-Based EMI Technique

**DOI:** 10.3390/s18051591

**Published:** 2018-05-17

**Authors:** Shuli Fan, Shaoyu Zhao, Baoxin Qi, Qingzhao Kong

**Affiliations:** 1 State Key Laboratory of Coastal and Offshore Engineering, Dalian University of Technology, Dalian 116024, China; shuli@dlut.edu.cn (S.F.); zhaoshaoyu916@163.com (S.Z.); 2 School of Civil Engineering, Shenyang Jianzhu University, Shenyang 110168, China; qibaoxin2005@163.com; 3 Department of Mechanical Engineering, University of Houston, Houston, TX 77004, USA

**Keywords:** structural health monitoring, piezoceramic transducer, electro-mechanical impedance, concrete damage, impact loading, damage monitoring

## Abstract

One of the major causes of damage to column-supported concrete structures, such as bridges and highways, are collisions from moving vehicles, such as cars and ships. It is essential to quantify the collision damage of the column so that appropriate actions can be taken to prevent catastrophic events. A widely used method to assess structural damage is through the root-mean-square deviation (RMSD) damage index established by the collected data; however, the RMSD index does not truly provide quantitative information about the structure. Conversely, the damage volume ratio that can only be obtained via simulation provides better detail about the level of damage in a structure. Furthermore, as simulation can also provide the RMSD index relating to that particular damage volume ratio, the empirically obtained RMSD index can thus be related to the structural damage degree through comparison of the empirically obtained RMSD index to numerically-obtained RMSD. Thus, this paper presents a novel method in which the impact-induced damage to a structure is simulated in order to obtain the relationship between the damage volume ratio to the RMSD index, and the relationship can be used to predict the true damage degree by comparison to the empirical RMSD index. In this paper, the collision damage of a bridge column by moving vehicles was simulated by using a concrete beam model subjected to continuous impact loadings by a freefalling steel ball. The variation in admittance signals measured by the surface attached lead zirconate titanate (PZT) patches was used to establish the RMSD index. The results demonstrate that the RMSD index and the damage ratio of concrete have a linear relationship for the particular simulation model.

## 1. Introduction

Collisions from moving vehicles can cause severe damage to column-supported concrete structures. From 1981 to 1990, 2418 bridges in the United States were hit by vessels [1]. Serious damage may result in a bridge collapse and cause serious consequences with regard to traffic operation and human life. One of the support piers of the Sunshine Skyway Bridge, located in Tampa Bay, Florida, USA, was collided with an empty 35,000 DWT bulk ship. This event caused severe casualties and significant economic losses due to the collapse of the bridge [2]. A recent disastrous event happened in 2007, when a freighter crashed into the Jiujiang Bridge in Guangdong, China, causing the collapse of more than 200 m of a bridge deck. More attention should be paid to the monitoring techniques to quickly assess the damage degree of bridge piers after a collision [3].

In recent years, lead zirconate titanate (PZT), as a smart material, has been widely used in the area of structural health monitoring (SHM) owing to its low cost, quick response, high stability, and dual sensing and actuation capacities. PZT sensors have been successfully applied in concrete structures to monitor the strength change, damage development, crack propagation, and impact location [4]. Based on PZT sensors, the electromechanical impedance (EMI) technique has emerged as a powerful and effective tool in SHM since Liang et al. [5] derived the one-dimensional EMI model in 1994. A large number of EMI-based experimental investigations have been conducted on concrete structures for crack detection [6,7,8], reinforced concrete corrosion monitoring [9,10], and concrete hydration monitoring [11,12,13]. Providakis et al. [14] carried out numerical simulations to detect damage of concrete beams using a PZT sensing system based on the electromechanical admittance (EMA) technique. Some experimental studies [15,16,17] highlighted the implementation of a network of PZT sensors based on the EMA technique concerning the damage detection and assessment of concrete beams under shear/flexural monotonic and cyclic loading. To quantitatively estimate the structural damage based on the EMI data, many damage metrics (DMs) or statistical indices were proposed and adopted, such as the root-mean-square deviation (RMSD), mean absolute percentage deviation (MAPD), and cross-correlation coefficient (CC) [18,19,20,21,22]. Sun et al. [23] used relative deviation to describe the structural damage. Wang et al. [24] determined the damage locations and severities of a concrete beam using the CC index. Some researchers [22,25] compared the performance of RMSD, MAPD, and CC as indicators of damage and pointed out that the RMSD and CC are suitable for evaluating the growth of damage based on the impedance spectra. However, CC has limitations to estimate the variation of a lab-sized steel truss bridge member under a temperature-varying environment. On the other hand, the differences between the baseline reading and the subsequent reading increase with the decrease of the CC metric value. Hu et al. [26] proposed a damage index *R_y_/R_x_* to predict the changes in amount of damage in reinforced concrete slabs, yet the values are small compared with that of RMSD. Therefore, most of the research utilizes RMSD-based tools to estimate the damage degree of concrete structures under different load types.

Practical applications of the RMSD index for damage evaluation require an understanding of the influence of the damage on the signal response of bonded PZT sensors. Narayanan and Subramaniam [27] decoupled the effect of stress and damage using the normalized conductance signature as a quantitative measurement and pointed out that the RMSD increases consistently with the increase of damage when the applied stress is removed. Lim et al. [28] found that the equivalent damping values increase with the increase of the damage severity and the RMSD response exhibits the equivalent damping change. Tseng and Wang [29] investigated the correlation of the RMSD index with the location and extent of damage based on numerical and experimental results. Soh et al. [30] quantified the damage on a prototype reinforced concrete bridge using RMSD in admittance signatures of the PZT patch during destructive load testing. Bhalla and Soh [31] provided an empirical calibration between identified stiffness and damage severity for a concrete structure. Liu et al. [32] monitored the freezing-thawing and the damage of a concrete structure based on the EMI technique. The damage evolution is reflected in the impedance spectra of the PZT sensors and the RMSD index increases with the freezing-thawing cycles and the crack depth. Yang et al. [33] studied the relationship between the distance of the PZT sensor location and the damage index. Ai et al. [34] used a slop-based RMSD index to detect concrete beam damage under impact loads. Karayannis et al. [35] quantified the damage level of a reinforced concrete beam using an RMSD index. All the above research indicates that the RMSD index can be used as an index for the estimation of the damage level in concrete structures. Although the RMSD index has a direct relationship with structural damage, quantitative evaluation of the damage degree is still difficult to achieve based on the conventional non-parametric approach.

This paper presents a novel method in which the impact-induced damage to a structure is simulated in order to obtain the relationship between the damage volume ratio to the RMSD index, and the relationship can be used to predict the true damage degree by comparison to the empirical RMSD. Collision damage of a bridge column by moving vehicles is simulated by using a concrete beam model subjected to continuous impact loadings by a freefalling steel ball. A series of numerical analyses are implemented to monitor the damage growth of the investigated model under impact loadings. The damage volume ratio obtained from the numerical results is utilized to quantify the damage degree of the numerical model before and after impact poundings. A linear relationship is found between the numerically-obtained RMSD index and the damage volume ratio for the particular simulation model. The proposed method could help better understanding of the damage degree for real structures by comparing the empirically-obtained RMSD index to the numerical results.

## 2. Theoretical Background

**Figure 1:**
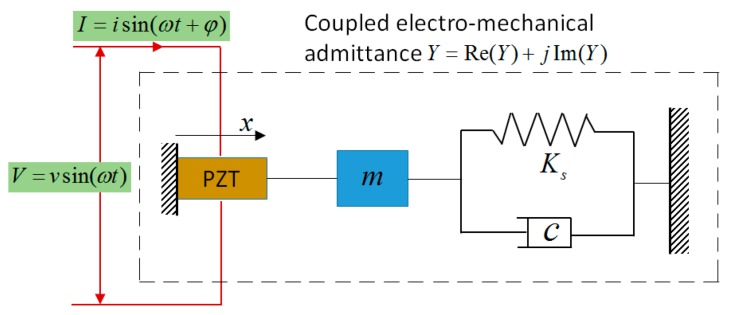
One-dimensional electro-mechanical system.

The EMI theoretical model was proposed by Liang et al. [36] and subsequently modified by many scholars [37,38,39,40]. The principles of the EMI-based damage monitoring technique employ both the direct and converse piezoelectric effect, in which one single PZT patch serves as both a sensor and an actuator, synchronously. The self-sensing property of piezoelectric material allows the PZT sensor to obtain frequency response functions between the input voltage and output current. Depending on the direct and converse effect of piezoelectric materials, the basis of the EMI technique is that the EMI signal received from the PZT sensor is directly related to the mechanical impedance of the host structure and is affected by the presence and growth of structure damage [34]. As shown in Figure 1, for a one-dimensional electro-mechanical system, the PZT sensor excites its host structure with a high-frequency band, and the impedance signature can be measured. The structural integrity can be qualitatively detected by sensing the electrical impedance or admittance of the piezoelectric transducers bonded on, or embedded into, the host structure and observing the changes to the signatures of measurement visually.

In the single degree-of-freedom mass-stiffness-damping system, the electrical admittance Y(ω) of a PZT patch is associated with the mechanical impedance of the host structure $Z_{s}\left(\omega \right)$ and that of the PZT patch $Z_{a}\left(\omega \right)$ for the frequency range of interest through the following equation [41]:

(1) $$Y=\frac{I_{o}}{V_{i}}=G\left(\omega \right)+jB\left(\omega \right)=j\omega \frac{wl}{h}\left\{\left(\frac{Z_{a}\left(\omega \right)}{Z_{a}\left(\omega \right)+Z_{s}\left(\omega \right)}\right){\bar{Y}}_{11}^{E}d_{31}^{2}\left(\frac{\tan\left(kl\right)}{kl}\right)+{\bar{\varepsilon }}_{33}^{T}-{\bar{Y}}_{11}^{E}d_{31}^{2}\right\}$$

where $V_{i}$ is the input voltage to the PZT actuator; $I_{o}$ is the output current from the PZT; w, l, and h denote the width, length, and thickness of the PZT patch, respectively; ω is the excitation frequency; $Z_{a}\;\mathrm{and}\;Z_{s}$ represent the mechanical impedances of the PZT patch and the host structure, respectively; $\bar{Y}{}_{11}^{E}=Y_{11}^{E}\left(1+\eta j\right)$ is the complex Young’s modulus of a PZT patch under a constant electric field, where η is the mechanical loss factor; $d_{31}$ is the piezoelectric constant of a PZT patch; the variable *k =* $\omega \sqrt{\rho /\bar{Y}{}_{11}^{E}}$ is the wave number and ρ is the density of a PZT patch; and $\bar{\varepsilon }{}_{33}^{T}=\varepsilon_{33}^{T}(1-\delta j)$ is the complex dielectric constant of a PZT patch under constant stress, where δ is the dielectric loss factor.

However, the above mentioned one-dimensional electro-mechanical system considers only the one-dimensional extensional actuation, ignoring the longitudinal actuations. Annamdas and Soh [42,43,44] presented the three-dimensional (3D) interaction of a transducer with the host structure, considering both the extensional and longitudinal actuations of the transducer. The complex electromechanical 3D directional sum admittance across the terminals of a PZT sensor for a surface bonded PZT and host structure can be expressed as [43]:

(2) $$\bar{Y}{}_{c}=\frac{2j\omega LW}{H}[\bar{\varepsilon }{}_{33}+Y_{R}\left(\begin{array}{c} d_{31}\lambda_{1}\left\{\left[A_{0}\sin kW-d_{31}\right]+R\left[C_{0}\sin kL-d_{32}\right]+R\left[E_{0}k\cos k2H-d_{33}\right]\right\}\\ +d_{32}\lambda_{2}\left\{R\left[A_{0}\sin kW-d_{31}\right]+\left[C_{0}\sin kL-d_{32}\right]+R\left[E_{0}k\cos k2H-d_{33}\right]\right\}\\ +d_{33}\lambda_{3}\left\{R\left[A_{0}\sin kW-d_{31}\right]+R\left[C_{0}\sin kL-d_{32}\right]+\left[E_{0}k\cos k2H-d_{33}\right]\right\} \end{array}\right)]$$

As Equations (1) and (2) show, the admittance signature gauged by a PZT patch is related not only to the mechanical impedance and material properties of the PZT patch, but also to the mechanical impedance of the host structure. Assuming that mechanical impedance and material properties of the PZT patch are constant, any changes in the mechanical impedance of the host structure are reflected in the admittance measurement. When concrete is damaged, the Young’s modulus of the concrete decreases, causing the modification in the structural stiffness. According to Equation (1), measurements of electrical admittance or impedance before and after damage can be qualitatively assessed.

## 3. Specimen and Materials

### 3.1. Finite Element Model of Beam Specimen

**Figure 2:**
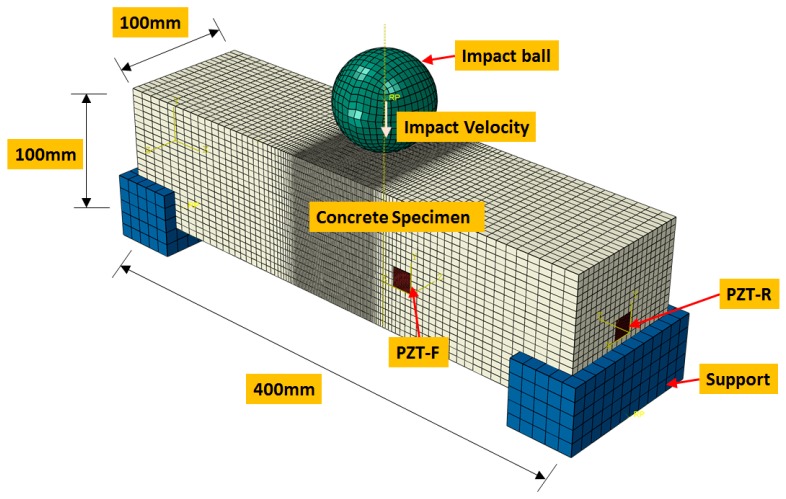
Finite element model of the impact test setup.

The three-dimensional finite element model is illustrated in Figure 2. The specimen as depicted in the figure has cross-sectional dimensions of 400 mm in length, 100 mm in width, and 100 mm in height, which is fixed on the rigid supports. In the numerical simulation, the concrete beam was impacted by a steel ball which was considered to be a rigid body. The ball with a mass of 1.4 kg dropped freely onto the top surface of the concrete beam at mid-span from the height of 1.25 m. The beam was subjected to 12 times of repeated impact loadings at the same falling height until the concrete beam fractured completely. Two PZT patches with a size of 15 mm × 15 mm × 0.5 mm (length × width × depth), were attached to the surface of the specimen to monitor the dynamic damage. One patch was bonded on the front surface of the concrete specimen (denoted as PZT-F) and the other one on the right surface of the beam (denoted as PZT-R). The material properties of PZT patch are listed in Table 1.

The finite element model consists of 62,416 nodes and 56,970 elements. The minimum element size is 1 mm and the maximum element size is 10 mm. The middle zone of the concrete beam was meshed with refined grids to simulate the damage procedure accurately. The supports, ball, and concrete beam are modeled using C3D8R elements, which are eight-node linear brick, reduced-integration elements for three-dimensional cases. The PZT patches were simulated using the eight-node linear piezoelectric elements (C3D8E) which are three-dimensional multi-physics coupled-field finite elements that couple electrical conduction with stress analysis. The supports and ball were modelled as steel. The steel material has Young’s modulus of 210 GPa, a Poisson’s ratio of 0.3, and a density of 7800 kg/m^3^. In order to simulate the contact behavior between the bottom point of the falling ball and the top surface of the concrete specimen, as well as the interfaces between the concrete beam and rigid supports, a general constraint algorithm that was frictionless in the tangential behavior and hard contact in normal behavior was used.

### 3.2. Concrete-Damaged Plasticity Model

The concrete material of the specimen utilized the plastic-damage constitutive model embedded in the software ABAQUS, which is a product of Dassault Systèmes Simulia Corp., Providence, RI, USA. The concrete-damaged plasticity model implemented in the software ABAQUS was based on the constitutive relationships proposed by Lublinear et al. [45]. To simulate cracking and crushing of concrete under dynamic or cyclic loadings, and this model was further amended by Lee and Fenves [46]. Based on basic damage mechanics, the effective stress $\bar{\sigma }{}_{ij}$ is defined as:

(3) $$\bar{\sigma }{}_{ij}=E_{ijkl}^{0}:\left(\varepsilon_{kl}-\varepsilon_{kl}^{p}\right)$$

where $E_{ijkl}^{0}$ is the initial elastic stiffness tensor, and $\varepsilon_{kl}$ and $\varepsilon_{kl}^{p}$ are the elastic strain tensor and plastic strain tensor, respectively.

Concrete experiences damage when the stress level reaches the failure stress. The damage causes the softening of the concrete material stiffness. Considering that concrete destruction is mainly caused by tensile damage, only the role of tensile damage was considered in this section. The damage can be expressed by the damage factor *d*. Accordingly, the relationship of Cauchy stress $\sigma_{ij}$ and effective stress $\bar{\sigma }{}_{ij}$ is defined by:

(4) $$\sigma_{ij}=\left(1-d\right)\bar{\sigma }{}_{ij}=\left(1-d\right)E_{ijkl}^{0}:\left(\varepsilon_{kl}-\varepsilon_{kl}^{p}\right)$$

The plastic flow potential function and yield surface make use of two stress invariants of the effective stress tensor, namely the hydrostatic pressure stress $\bar{p}$:

(5) $$\bar{p}=-\frac{1}{3}\bar{\sigma }{}_{ij}:I$$

and the Mises equivalent effective stress $\bar{q}$:

(6) $$\bar{q}=\sqrt{\frac{3}{2}(\bar{S}{}_{ij}:\bar{S}{}_{ij})}$$

where $\bar{S}{}_{ij}$ is the effective stress deviator, defined as:

(7) $$\bar{S}{}_{ij}=\bar{\sigma }{}_{ij}+\bar{p}I$$

As for as effective stresses, the yield function equation takes the following form:

(8) $$F=\frac{1}{1-\alpha }(\bar{q}-3\alpha \bar{p}+\beta \left(\varepsilon^{p}\right)\langle \hat{\bar{\sigma }}{}_{max}\rangle -\gamma \langle -{\hat{\bar{\sigma }}}_{max}\rangle )-{\bar{\sigma }}_{c}\left(\varepsilon_{c}^{p}\right)=0$$

(9) $$\gamma =\frac{3\left(1-K_{c}\right)}{2K_{c}-1}$$

where α and β are dimensionless material constants, ${\hat{\bar{\sigma }}}_{max}$ is the maximum principal effective stress, $K_{c}$ is the strength ratio of concrete under equal biaxial compression to triaxial compression, and ${\bar{\sigma }}_{c}\left(\varepsilon_{c}^{p}\right)$ is the effective compressive cohesion stress.

The concrete damage plasticity model presumes non-associated potential plastic flow:

(10) $${\dot{\varepsilon }}_{ij}^{p}=\dot{\lambda }\frac{\partial G\left({\bar{\sigma }}_{ij}\right)}{\partial {\bar{\sigma }}_{ij}}$$

The flow potential *G* used for this model is the Drucker-Prager hyperbolic function:

(11) $$G=\sqrt{{\left(\in \sigma_{t0}\tan\psi \right)}^{2}+{\bar{q}}^{2}}-\bar{p}\tan\psi$$

where $\psi \left(\theta ,f_{i}\right)$ is the dilation angle measured in the p-q plane at high confining pressure, $\sigma_{t0}$ is the uniaxial tensile stress at failure, and ϵ is a parameter, referred to as the eccentricity, that defines the rate at which the function approaches the asymptote (the flow potential tends to a straight line as the eccentricity tends to zero).

**Figure 3:**
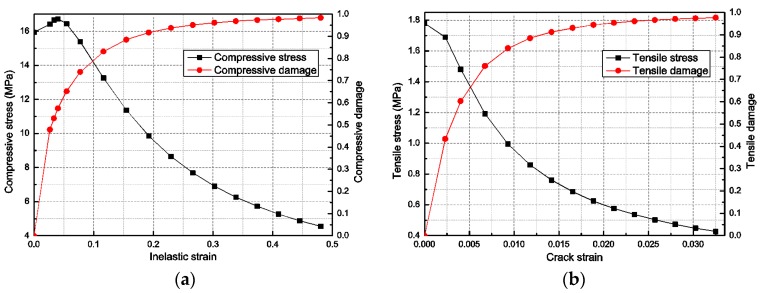
Concrete material properties: (**a**) compressive properties; and (**b**) tensile properties.

The main mechanical properties of concrete used in this paper were: average density ρ = 2500 kg/m^3^, average compressive strength $f_{c}$ = 16.7 MPa, the direct tensile strength $f_{t}$ = 1.80 MPa, Poisson coefficient γ = 0.20 and elastic modulus E = 28 GPa. Figure 3a,b show the compressive and tensile properties of concrete.

## 4. Numerical Results and Analysis

### 4.1. Damage Procedure of the Concrete Specimen under Impact Loads

**Figure 4:**
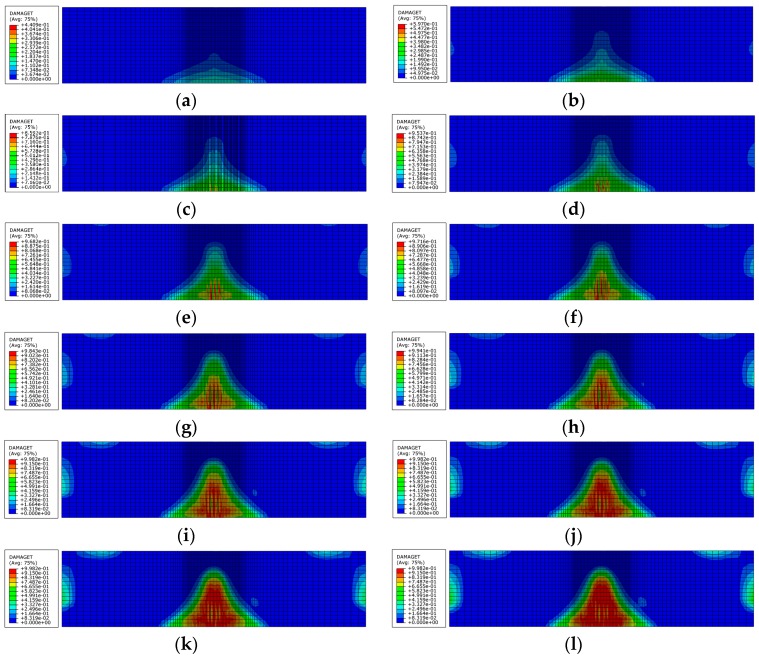
Damage of concrete specimen at different impact times: (**a**) first impact; (**b**) second impact; (**c**) third impact; (**d**) fourth impact; (**e**) fifth impact; (**f**) sixth impact; (**g**) seventh impact; (**h**) eighth impact; (**i**) ninth impact; (**j**) 10th impact; (**k**) 11th impact; and (**l**) 12th impact.

Damage factor is a commonly-used index to present the damage of concrete material, which is defined as the scalar stiffness degradation in ABAQUS software. The impact damage mode of the specimen using the concrete plastic-damage constitutive model was derived from the numerical simulation, as shown in Figure 4. The minor differences in damage in the contour profile can be ignored and the concrete-damaged plasticity model can be used to simulate the impact damage of the concrete. Two PZT patches were employed for tracking the initial damage emergence and growth due to the stiffness degradation of concrete with the increase of impact times.

Figure 4 illustrates various tensile damage contour plots of concrete beam at different impact times. A tensile damage value of 0 shows the elastic behavior of concrete. More specifically, the values from 0 to 0.7 denote the formation of micro-cracks that are very small and invisible; the values from 0.7 to 1 indicate that macro-cracks emerge in the concrete; a tensile damage value of 1 means complete concrete failure. At the first impact loading, some slight damage starting from the bottom of the specimen appeared, but the initial damage of the concrete structure, as shown in Figure 4a,b, is invisible and undiscoverable by the naked eye. In order to ensure the safety and stability of the structure, it is important for us to discover in a timely manner the incipient damage generation and guard against the damage propagation. With the increase in impact times, the crack and damage gradually grew to the top surface along the central axis of the concrete beam and local damage in the mid-span of the beam became obvious. As seen from Figure 4i, the beam could be considered broken along with the twelfth times’ impact load application.

### 4.2. EMI Response Spectra Analysis

**Figure 5:**
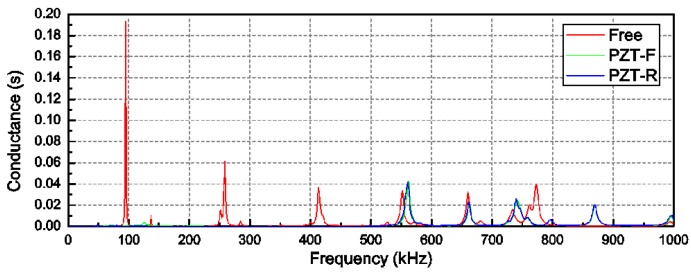
Conductance spectra between the free condition and the interaction condition.

In order to ensure the detection sensitivity of the EMI-based technique, the conductance (real part of the admittance) was scanned with a wide sweep range from 1 kHz to 1 MHz to confirm a suitable frequency range at the beginning of the numerical experiment. It is obvious that the conductance signature has a more direct relation to the resonant frequency than admittance signature and susceptance signature. This is the reason why conductance was chosen for this scan. The test results of the conductance spectra are plotted in Figure 5. The red line is the conductance spectra when the PZT patch was free in the air, whereas the green line and blue line are the signatures when the PZT patches were bonded on the front surface and right surface, respectively. In the free condition, the first six resonant frequencies of the PZT patch were excited. In the interaction condition between PZT patches and the host structure, the conductance amplitudes at the first three resonant frequencies were significantly suppressed. However, the fourth resonant frequency shifted from 550 kHz to 560 kHz, and the conductance peaks slightly increased at the same time when the PZT patches changed to the interaction condition from the free condition. This is because the bonding of PZT increased the system stiffness. The result indicates that the excitation signal at the frequency point of approximately 560 kHz was sensitive to the structure mechanical properties changes. Therefore, a frequency range from 550 kHz to 580 kHz was chosen as the sweep frequency band for the scanning of the impedance and admittance spectra during the EMI-based impact loading damage detection test.

**Figure 6:**
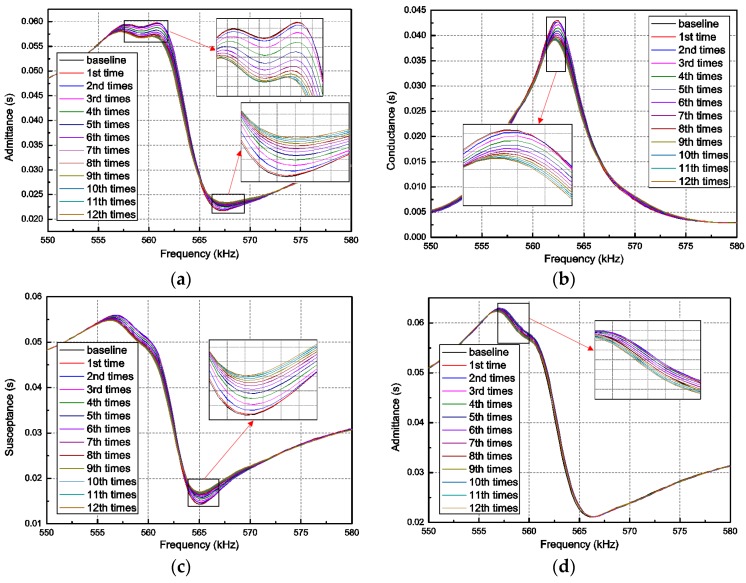
Admittance signatures in different impact load case: (**a**) Admittance of PZT-F; (**b**) conductance of PZT-F; (**c**) susceptance of PZT-F; and (**d**) admittance of PZT-R.

Figure 6 shows the electrical admittance signatures of the PZT-F patch and PZT-R when the concrete structure was subjected to 12 times the continued impact force. Not only the admittance spectra, but also the spectra for its real (conductance) and imaginary (susceptance) parts, were measured over the frequency range between 550 kHz and 580 kHz. Compared with these EMI curves, it is encouraging to see the changes in all these signatures correspond with the impact times. Clearly, the deviation of the spectra grows with the increasing of impact times. The reason is that the damage was increasingly serious as the steel ball gradually impacted the concrete specimen. As can be found from the definition and derivation of the concrete plastic damage model, the development of damage caused a stiffness degradation of the concrete. The mechanical impedance of the structure altered due to the stiffness degeneration of concrete. As a result, the coupling admittance spectra between PZT-F and the host structure shifted, as shown in Figure 6a–c.

The admittance signatures of PZT-R (the one bonded on the right surface) are shown in Figure 6d. It can be seen that there are only some very slight and irregular variations between these signatures during 12 times the impact loading and baseline as compared to the results from PZT-F. This can be explained by the phenomenon that PZT-R was located on the right surface of the concrete specimen, which is further away from the primary region of the dynamic damage evolution. It is well-known that the sensing zone of the PZT patch is restricted to a small region and sensing sensitivity decreases with the increase of the distance between the damage area and the PZT location. It should be noted that the PZT impedance signature is highly influenced by the location and type of the PZT sensor. The sensitivity of the results decreases with the increase of distance between the damage and the sensor. As shown from the numerical results, the impedance of the PZT-R sensor, which is far away from the damage area, is not as sensitive as the other one that is near the crack. Actually, this is a common disadvantage of impedance-based methods because the impedance change is mainly determined by the change of structural local stiffness (approximately 0.5 m in radius direction). To improve the sensing range using this type of method, many researchers deployed multiple sensors at the locations of interest for structural health monitoring. In addition, the admittance signatures measured by PZT-R was also affected by the damage occurring in the right surface of the beam.

### 4.3. Measures of Damage Quantification

**Figure 7:**
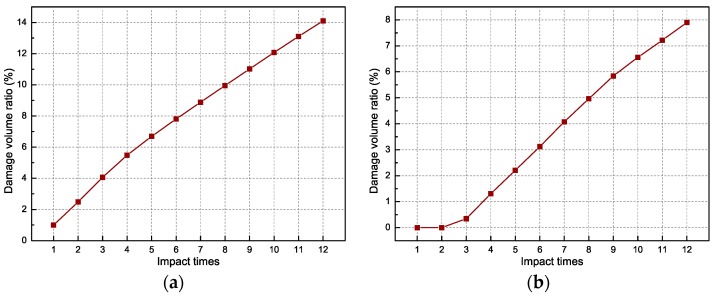
Damage volume ratio of concrete beam: (**a**) global damage (*d* > 0); and (**b**) local damage (*d* > 0.7).

To quantitatively depict the emergence and development of the damage of a concrete beam under impact loading, the damage volume ratio (DVR), as an evaluating indicator of the concrete structure, is proposed, which can be calculated by the weighted average method given as:

(12) $$DVR\left(\%\right)=\sum_{i=1}^{i=N}d_{i}^{t}V_{i}/\sum_{i=1}^{i=N}V_{i}\times 100$$

where i denotes the ith element in the finite element model, N is the total number of elements, and $d_{i}^{t}$ and $V_{i}$ are the tensile damage factor and volume of the ith element in the model, respectively. Figure 7a plots the global damage volume ratio with the increasing of impact times. As can be seen, the damage volume ratio grows linearly with the increase of the impact times. To further understand the concept, the damage volume ratio with a damage factor *d* > 0.7 was used to depict the local damage of the concrete beam, which can be observed in Figure 7b. The damage factor 0.7 is a crucial threshold which demonstrates the severe damage in the mid-span of the concrete beam. After the third impact loading acted on the beam, the DVR started to increase linearly. This denotes that wider cracks begin to emerge due to the concrete yield.

The RMSD index is used to calculate the difference between values of baseline measurement of the admittance signature and the signature in the different damage status. As mentioned above, it can be used as a damage indicator to quantitatively estimate the difference in the admittance signatures before and after the damage. The mathematical expression of the metric in terms of the real part of the electrical admittance Re(Y) of the attached PZT patch is given in [18] as the following function:

(13) $$RMSD={\left(\overset{n}{\underset{i=1}{\sum }}{\left(Y_{i}^{k}-Y_{i}^{0}\right)}^{2}/\overset{n}{\underset{i=1}{\sum }}{\left(Y_{i}^{0}\right)}^{2}\right)}^{1/2}$$

where $Y_{i}^{0}$ represents the reference admittance signature of the PZT at the *i*th measurement point, and $Y_{i}^{k}$ represents the corresponding admittance signature at the *i*th measurement point for each *k*th load case.

### 4.4. Empirical Fitting Curves for Predicting Damage Levels

**Figure 8:**
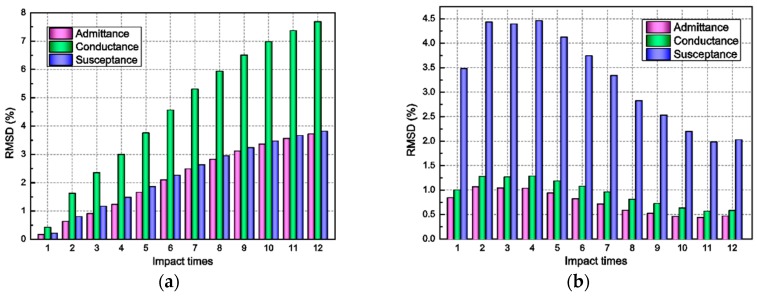
The RMSD damage metrics of the admittance signatures: (**a**) RMSD changes of PZT-F with impact times; and (**b**) RMSD changes of PZT-R with impact times.

All of these parameters’ variations and analyses in the previous section demonstrate the impact damage evolution to some extent. However, the results from the conductance signatures are, so far, qualitative. To account for the variations in the admittance curves during the repeated impact loading, the RMSD index is applied to evaluate the impact damage quantitatively, as shown in Figure 8. For the results of PZT-F, the statistical indicator RMSD for these parameters shows a similar trend that was increasing with the repeated impact, illustrating that there was increasing damage inside the concrete sample. The RMSD metric for conductance (the real part of admittance) shows the highest sensitivity and the rest are of low sensitivity. As observed in Figure 8a, the RMSD of the conductance has the higher percentage in the course of impact, which almost double those values of the other two parameters. Nevertheless, the RMSD index of the admittance signatures obtained by PZT-R does not present the distinct rule along with the impact times, as displayed in Figure 8b.

**Figure 9:**
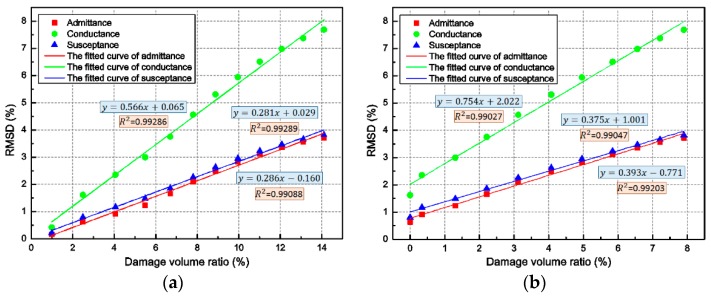
The RMSD based damage volume ratio: (**a**) global damage (*d* > 0); and (**b**) local damage (*d* > 0.7).

In order to further associate the RMSD of admittance signatures with the damage level of the concrete beam, the RMSD-based damage volume ratio is proposed. The two kinds of damage volume ratio, global damage, and local damage, are chosen to build the relationship with the RMSD of the admittance signals of PZT-F, as depicted in Figure 9. According to the distribution characteristics of these key points, the linear relations were fitted. By observing the pictures, the fitted relationship curves have a good fitting degree, which is more than 0.99. Take the green line from the Figure 9a as an example: if the RMSD of conductance signal was obtained, the damage volume ratio can be determined using the formula y=0.566x+0.065. In this way, the global damage degree of the concrete beam can be determined.

To further understand the relationship between the RMSD of the admittance signatures and the damage level of the concrete beam, the RMSD-based damage volume ratio is proposed. As discussed above, the damage value of 0.7 is a crucial threshold which demonstrates the emergence of slight damage and the initial visible crack of the concrete. Thus, the damage volume ratio for *d* > 0.7 was chosen to build relationships with the RMSD of admittance signatures of PZT-F, as depicted in Figure 9b. According to the characteristics of the RMSD-damage volume ratio curves, the RMSD-based damage volume ratio metric is able to clearly quantify the variation of admittance signatures due to the development of the concrete damage and conveniently assess the damage level of the concrete beam under impact loading. Empirically, the local damage level of the concrete beam can be worked out using the linear relationship expressions in Figure 9b. Consequently, the RMSD-based damage volume ratio metrics are able to intuitively assess and predict the damage level of the concrete beam under impact loading. More specifically, for a general case or an experimental study, the detailed procedure to estimate the damage degree of the specimen or structure is as follows: (1) build the numerical model (including surface-mounted PZT sensors) of the specimen or structure, and then build the relationship (fitting curve) between the numerically-obtained RMSD index and damage volume ratio via simulation results; and (2) use the numerically-obtained relationship to predict the true damage degree of the specimen or structure by comparison to the experimentally-obtained RMSD index.

### 4.5. Verification of the Fitted Curves

**Figure 10:**
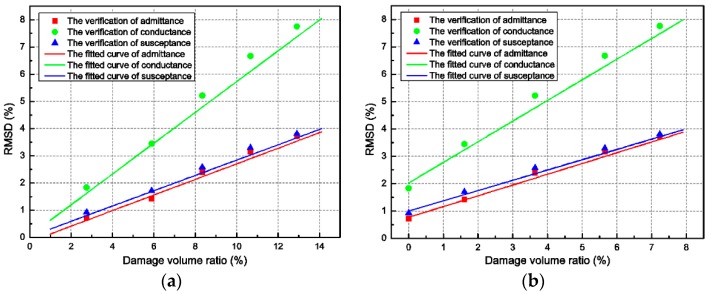
Verification of the fitted curves: (**a**) global damage (*d* > 0); and (**b**) local damage (*d* > 0.7).

To verify the accuracy of the fitted equation in Figure 9, the damage volume ratio of the concrete beam should be derived at any measured RMSD of the admittance signals under different levels of impact load. In this section, the same beam model as mentioned in the above section was impacted by the steel ball with falling height of 1.5 m. The RMSD and the damage volume ratio were measured when the impact times were 2, 4, 6, 8, and 10. The obtained results are compared with the fitted curves, as shown in Figure 10.

The verification results are found to be in good agreement with the fitted curves from Figure 10. Hence, the effectiveness of the fitted equation in Figure 9 has been demonstrated. In addition, the difference between the verification results and the fitted curves might be caused by the limitation of the concrete constitutive model, numerical errors, and the low correlation coefficients of the fitted equations.

## 5. Conclusions

In this paper, the applicability and feasibility of an EMI-based damage monitoring technique for impact damage detection in the concrete structure was investigated. Numerical simulations of impact events on a concrete beam using the concrete damaged plasticity constitutive model implemented in ABAQUS was performed. There is a general agreement that the damage volume ratio of concrete can be estimated using the RMSD index based on changes of the electro-mechanical impedance signatures. Thus, the alterations in the admittance or impedance spectra at different impact times are denoted to be an indicator of the impact damage. All the numerical results demonstrate that the statistical damage metric RMSD-based damage volume ratio can be used to quantify the variations in the signatures of admittance and evaluate the damage degree of the concrete beam. According to the linear relationship between the damage volume ratio and the RMSD index, the EMI-based approach shows promising results for estimating the impact damage of a concrete structure through inverse computation based on the RMSD index. It has remarkable potential to assess the damage level and health condition of a concrete structure. In future works, the influence of the distance between the sensor and damage zone on the sensitivity and accuracy of the results will be studied. In addition, the concepts developed herein will be experimentally investigated to the monitoring of the concrete piers in a real environment.

## Figures and Tables

**Table 1:** Material properties of the PZT patch used in the numerical study.

Physical Parameters		Values
Young’s modulus at constant electric field	$Y_{11}^{E}$	4.6 × 10^10^ N/m^2^
Poisson ratio	ν	0.3
Density	ρ	7500 kg/m^3^
Piezoelectric strain coefficient	*d*_31_, *d*_32_	−274 × 10^12^ m/V
	*d*_33_	593 × 10^12^ m/V
	*d*_15_, *d*_24_	741 × 10^12^ m/V
Electric permittivity at constant stress	$\varepsilon_{11}^{T}$, $\varepsilon_{22}^{T}$	1.505 × 10^−8^ F/m
	$\varepsilon_{33}^{T}$	1.301 × 10^−8^ F/m
Dielectric loss factor	δ	0.012
Mechanical loss factor	η	0.001
